# Vaccination of Mice with *Salmonella* Expressing VapA: Mucosal and Systemic Th1 Responses Provide Protection against *Rhodococcus equi* Infection

**DOI:** 10.1371/journal.pone.0008644

**Published:** 2010-01-13

**Authors:** Aline F. Oliveira, Luciana P. Ruas, Silvia A. Cardoso, Sandro G. Soares, Maria-Cristina Roque-Barreira

**Affiliations:** Departamento de Biologia Celular e Molecular e Bioagentes Patogênicos, Faculdade de Medicina de Ribeirão Preto, Universidade de São Paulo, Ribeirão Preto, São Paulo, Brazil; BMSI-A*STAR, Singapore

## Abstract

Conventional vaccines to prevent the pneumonia caused by *Rhodococcus equi* have not been successful. We have recently demonstrated that immunization with *Salmonella enterica* Typhimurium expressing the VapA antigen protects mice against *R. equi* infection. We now report that oral vaccination of mice with this recombinant strain results in high and persistent fecal levels of antigen-specific IgA, and specific proliferation of the spleen cells of immunized mice in response to the *in vitro* stimulation with *R. equi* antigen. After *in vitro* stimulation, spleen cells of immunized mice produce high levels of Th1 cytokines and show a prominent mRNA expression of the Th1 transcription factor T-bet, in detriment of the Th2 transcription factor GATA-3. Following *R. equi* challenge, a high H_2_O_2_, NO, IL-12, and IFN-γ content is detected in the organs of immunized mice. On the other hand, TNF-α and IL-4 levels are markedly lower in the organs of vaccinated mice, compared with the non-vaccinated ones. The IL-10 content and the mRNA transcription level of TGF-β are also higher in the organs of immunized mice. A greater incidence of CD4^+^ and CD8^+^ T cells and B lymphocytes is verified in vaccinated mice. However, there is no difference between vaccinated and non-vaccinated mice in terms of the frequency of CD4^+^CD25^+^Foxp3^+^ T cells. Finally, we show that the vaccination confers a long-term protection against *R. equi* infection. Altogether, these data indicate that the oral vaccination of mice with *S. enterica* Typhimurium expressing VapA induces specific and long-lasting humoral and cellular responses against the pathogen, which are appropriately regulated and allow tissue integrity after challenge.

## Introduction

*Rhococcus equi*, a gram-positive bacterium, is a facultative intracellular coccobacillus that causes bronchopneumonia in foals aged 1 to 6 months. It has also been increasingly identified as an opportunistic pathogen of immunocompromised humans [1], such as those with acquired immunodeficiency syndrome (AIDS) or undergoing immunosuppressive therapy [2], [3]. First isolated from pulmonary lesions of foals by Magnusson in 1923 [4], *R. equi* is able to infect, survive, and multiply inside the host cells, mainly in alveolar macrophages [5]. The infection begins through inhalation of bacteria from the soil or dust and can result in a severe disease, characterized by chronic pyogranulomatous pneumonia and lung abscesses in both foals and humans. Extrapulmonary lesions may also occur [1].

Although the pathogenic mechanisms of *R. equi* remain largely unknown, there is evidence that virulent strains contain a large 85- to 90-kb plasmid bearing a 27.5-kb pathogenicity island that encodes, among others, nine genes of the virulence-associated protein (vap) family [6], [7]. One member of this family is VapA, a highly immunogenic 15–17 kDa protein that is abundantly expressed on the bacterial surface [6], [8] and plays a crucial role in pathogen growth inside macrophages as well as disease development [9], [10]. Furthermore, VapA is thought to be important in generating immunity against *R. equi* [11], [12].

Several vaccination strategies have been assayed in an attempt to prevent rhodococcosis. However, there are currently no safe and effective vaccines against the disease, and the only method to avoid that foals of an endemic farm develop *R. equi* pneumonia is the administration of specific hyperimmune plasma [13], which can provide positive effects [14] but is expensive, labor-intensive, and not universally effective [15], [16]. Therefore, an effective vaccine suitable for large-scale administration is greatly needed for the prevention of rhodococcal infection.

To protect host against rhodoccocosis, a vaccine may need to stimulate both cell-mediated and humoral immunity [14]. Data obtained from immune adult horses and deepened by studies in the murine model of rhodococcosis indicate that resistance to *R. equi* is mainly mediated by T-lymphocyte and depends on IFN-γ production [14], [17]–[19].

In recent years, several studies have demonstrated the feasibility of using attenuated Gram-positive and Gram-negative intracellular bacteria as live vectors for the oral delivery of recombinant vaccine antigens [20], [21]. Several *Salmonella enterica* Typhimurium strains submitted to attenuation procedures lost their pathogenicity but remained invasive and are used as live vectors for delivery of foreign antigens. These strains are able to induce protective mucosal, humoral, and systemic immune responses against bacteria, viruses, and parasites in a variety of animal models [22], [23]. When used as oral vehicle, they invade enterocytes of the small intestine, including the M cells of the Peyer's patches, before disseminating to the mesenteric lymph nodes and through the reticuloendothelial system to deep tissues, such as the liver and spleen. Both antibody and cellular specific responses to recombinant antigens expressed by *Salmonella* strains have been detected after immunization of mice via mucosal surfaces [24], [25]. The response includes the production of specific secretory immunoglobulins [25], [26].

We have previously reported that oral vaccination of mice with an attenuated *S. enterica* Typhimurium vaccine strain expressing the VapA protein confers protection against virulent *Rhodococcus equi* [27]. In the present work we examined the profile of the immune response that was developed in vaccinated mice and whether the immunization procedure was able to induce a long-term protection against *R. equi* infection.

## Materials and Methods

### Experimental Animals

Each experimental or control group consisted of five BALB/c mice, which were housed under specific-pathogen-free conditions in the Animal Research Facilities of the Medical School of Ribeirão Preto-USP. All animals used for the experiments were female, at 6 to 8 wk of age. The Ethics Committee on Animal Research of the University of São Paulo approved all the procedures performed in the studies described here.

### Bacterial Strain and Growth Conditions

The *S. enterica* Typhimurium χ3987 *Δcya Δcrp Δasd* attenuated strain [28] was employed previously by us for expression of the VapA antigen [27]. Bacterial strains were grown in Luria Broth (LB) medium, in a rotary shaker at 250 rpm, at 37°C. For the preparation of bacterial suspensions for administration in mice, overnight cultures of the *S. enterica* Typhimurium χ3987 strains were precipitated by centrifugation (3000×g; 15 min), and the pellet was re-suspended in phosphate-buffered saline (PBS) to a final cell density of 1-5×10^10^ CFU/mL. CFU values were determined by plating dilutions of the bacterial suspension onto MacConkey agar plates.

The virulent strain of *R. equi* ATCC33701 was kindly provided by Dr. Shinji Takai (University of Kitasato, Towada, Japan). *R. equi* was inoculated into 250 mL brain heart infusion broth (BHI, Oxoid, Hampshire, England). Cultures were incubated in a rotary shaker at 100 rpm, 37°C, for 60 h (optical density, OD_600 = _1.3). For inoculation into mice, bacterial cultures were washed and resuspended in PBS; actual numbers of inoculated bacteria were confirmed by plating serial dilutions on BHI agar plates at the injection time.

### Protocol of Mice Immunization

On days 0 and 14, groups of BALB/c mice were intragastrically immunized by gavage needle with 1×10^9^ CFU of either *Salmonella enterica* Typhimurium χ3987-pYA3137*vapA* or *Salmonella enterica* Typhimurium χ3987-pYA3137 re-suspended in 200 µL PBS. In a 3^rd^ group, mice were inoculated with 200 µL PBS only.

Throughout this work, mice receiving *S. enterica* Typhimurium χ3987-pYA3137*vapA* were denominated immunized mice and those inoculated with either *S. enterica* Typhimurium χ3987-pYA3137 or PBS were denominated control mice.

### Estimation of Bacteria in the Organs

The number of virulent *R. equi* recovered from the organs of mice following intravenous inoculation was estimated on day 8 post-infection. Briefly, groups of mice were infected with a sublethal dose (4×10^6^ CFU) [27] of virulent *R. equi*, 14 days after the last immunization, and euthanized by cervical dislocation. Lung, spleen and liver were collected, aseptically weighed, homogenized, and serially diluted in PBS, to determine the number of CFU. Aliquots of 100 µL homogenates were plated onto BHI agar in duplicates. The plates were incubated at 37°C for 36 h, for bacterial counting.

To evaluate long-term protection, the challenge of mice was done 5 months after the last immunization, using the same dose of *R. equi*.

### Detection of Specific IgA Antibody

Samples of feces were collected from five mice per group on days 7, 14, 21, 28, 35, and 42 after the 2^nd^ immunization and assessed by enzyme-linked immunosorbent assay (ELISA) for IgA detection. After R. equi challenge, lung samples of similar weight were harvested from immunized and control mice, and homogenized in 1 mL PBS. The supernatant of each sample was also assessed for IgA concentration. Briefly, 96-well microtitre plates were coated overnight at 4°C with 1 µg/well APTX (acetone precipitate containing surface proteins of R. equi - a VapA-enriched antigen preparation) diluted in 0.2 M carbonate buffer (pH 9.6). Individual sample were added at a dilution of 1∶20 in PBS-1% gelatin, and the plates were incubated for 2 h at 37°C. Rabbit anti-mouse IgA antibody was added at a 1∶500 dilution, and the plates were incubated for 1 h at 37°C. Goat anti-rabbit antibody conjugated with horseradish-peroxidase (Sigma) was then added at 1∶1,000, and the plates were incubated for another hour at 37°C. Color was developed for 15 min at 37°C with 3,3′,5,5′-tetramethylbenzidine substrate (TMB) prepared according to the manufacturer's instruction (Pierce Chemical Co., Rockford, IL, USA), and the reaction was stopped with 2 M H_2_SO_4_ before readings were taken at 450 nm in a Microplate Scanning Spectrophotometer (PowerWave X, Bio-Tek Instruments, Inc., Winooski, VT, USA).

### Proliferation Assay

Immunized and control mice were euthanized 14 days post-infection and their spleens were removed. The spleen cell suspensions were washed in RPMI 1640 medium (Invitrogen) and treated for 5 min with erythrocyte lysing buffer (9 volumes of 0.16 M NH_4_Cl and 1 volume of 0.17 M Tris-HCl, pH 7.5). The erythrocyte-free cells were then washed three times and adjusted to 2×10^6^ cells/mL in RPMI 1640 containing 5% fetal bovine serum (FBS), and supplemented with penicillin G, streptomycin, and amphotericin B (1×10^4^ U/mL, 1×10^4^ µg/mL, and 25 µg/mL, respectively). The cell suspension (1 mL) was distributed in a 24-well tissue culture plate (Corning) and cultured in the presence of APTX (5 µg/mL), Concanavalin A (Sigma) (2 µg/mL), or medium alone, at 37°C, in a humidified atmosphere with 5% CO_2_ for 72 h. After 60 h of incubation, 3H-thymidine (0.5 µCi) was added to each well under sterile conditions. The cultures were kept under similar conditions for a further 12 h; thereafter the cells were harvested and the radioactivity was measured by suspending the harvested cells in Bray's scintillation fluid and counting on a β-counter (Rackbeta, 1214). The results are expressed as counts per minute (c.p.m.).

### Cytokine Detection in Culture Supernatants of Spleen Cells

Spleen cells from immunized and control mice, aseptically removed 14 days post-infection, were prepared as described in the previous item. A concentration of 2×10^6^ cells/mL was distributed in a 24-well tissue culture plate (Corning), and cultured in the presence of APTX (5 µg/mL), LPS (Sigma) (1 µg/mL) plus IFN-γ (BD Pharmingen) (1 ng/mL) or medium alone, at 37°C, in a humidified 5% CO2 incubator. The culture supernatants were collected after 48 h for determination of IL-12, IFN-γ, TNF-α, IL-10, and IL-4 cytokines, using a sandwich ELISA, according to the manufacturer's instructions (BD Pharmingen). Cytokine concentrations were determined by comparison with standard curves constructed with known amounts of the respective mouse recombinant cytokines.

### Detection of Cytokines, Nitric Oxide, and Hydrogen Peroxide in the Organs Homogenates

Lung, liver, and spleen were harvested from immunized and control mice on days 2, 4, 8, and 10 post-infection, and homogenized in 1 mL PBS using a tissue homogenizer. The samples were centrifuged at 5000×g for 10 min, and the supernatants were stored at - 20°C until assay.

The levels of IL-12p40, IFN-γ, TNF-α, IL-10, and IL-4 in the organs homogenates were quantified using a commercially available kit, according to the manufacturer's instructions (OptEIA set; Pharmingen, San Diego, CA, USA). The cytokines concentrations were determined by comparison with standard curves generated using known amounts of the respective mouse recombinant cytokines. The lower limits of detection were 15 pg/mL for IL-12p40 and TNF-α, 30 pg/mL for IFN-γ and IL-10 , and 7 pg/mL for IL-4.

NO production was quantified by accumulation of nitrite in the homogenates of lung, liver, and spleen of mice, using the standard Griess reaction. Briefly, 50 µL organ homogenates were incubated with an equal volume of the Griess reagent (1% sulfanilamide, 0.1% naphthyl ethylenediamine dihydrochloride, 2.5% H_3_PO_4_) for 10 min, at room temperature. The absorbance was measured at 550 nm in the Microplate scanning spectrophotometer. The conversion of absorbance into micromolar concentrations of NO was deduced from a standard curve using a known concentration of NaNO_2_.

The H_2_O_2_ levels in the organs homogenates were measured by horseradish peroxidase-dependent phenol red oxidation methods as previously described [29].

### Real-Time Quantitative PCR Analysis

Total RNA was isolated from the spleen cells of immunized and control mice using the TRIzol Reagent (Invitrogen Life Technologies, Carlsbad, CA, USA), following the manufacturer's instructions. cDNA synthesis was performed in a final volume of 20 µL, using ImProm-II Reverse Transcriptase (Promega Corporation, Madison, WI, USA). The reaction mixture contained 4 µg of total RNA, 20 pmol of oligo dT primer (Invitrogen Life Technologies), 40 U RNAsin, 1 M dNTP mix, and 1 U reverse transcriptase buffer. The cDNA was treated with 10 µg of RNase (Gibco, Carlsbad, CA, USA) and then immediately used or stored at - 20°C. PCR amplification and analysis were achieved using an ABI Prism 7500 sequence detector (Applied Biosystems, Foster City, CA, USA). All the reactions were performed with SYBR Green Master Mix (Applied Biosystems) using a 20 µL volume in each reaction, which contained 2 µL of template cDNA, 5 pmol of each primer, and 12.5 µL of SYBR Green. Data were normalized by β-actin gene, and the relative quantification was carried out by the delta Ct method (Applied Biosystems, Foster City, CA, USA). The primers used for PCR amplification were as follows: for GATA-3, 5′- AAGAAAGGCATGAAGGACGC -3′ (forward) and 5′- GTGTGCCCATTTGGACATCA -3′ (reverse); for T-bet, 5′- CACTAAGCAAGGACGGCGAA -3′ (forward) and 5′-CCACCAAGACCACATCCACA -3′ (reverse); TGF-β 5′- GACTCTCCACCTGCAAGACCA -3′ (forward) and 5′- GGGACTGGCGAGCCTTAGTT -3′ (reverse); for β-actin, 5′- AGCTGCGTTTTACACCCTTT -3′ (forward) and 5′- AAGCCATGCCAATGTTGTCT -3′ (reverse).

### Flow Cytometry Analysis

Spleen cells were harvested from immunized and control mice 8 days post-infection. The cells were washed with ice-cold PBS and incubated for 30 min at 4°C with 0.5 µg anti-CD16/CD32 mAb (Fc block, clone 2.4G2, BD Pharmingen, San Diego, CA), followed by addition of 0.5 µg per 1×10^6^ cells of the antibodies. Phycoerythrin-conjugated anti-CD19, anti-CD4, and anti-CD8, and fluorescein isothiocyanate-conjugated anti-CD3 and anti-CD25 mAbs were used for surface staining. Intracellular staining for Foxp3 was done with PeCy5-conjugated anti-mouse Foxp3, according to the manufacturer's instruction (all from BD Pharmingen). After 45 minutes of incubation on ice and washing with PBS, cells were fixed and analyzed on FACScan flow cytometer (BD Bioscience).

### Statistical Analysis

Statistical analysis was performed using analysis of variance followed by the parametric Tukey-Kramer test (INSTAT software, GraphPad, San Diego, CA, USA). Results are presented as the mean and SEM. A *P* value<0.05 was considered statistically significant. A two-way ANOVA was used for repeated measurements.

## Results

### Vaccination with *Salmonella* χ3987-pYA3137*vapA* Protects Mice against *R. equi* Infection and Elicits Strong Mucosal Humoral Response and Systemic Cell Immunity

Eight days after infection with *R. equi*, BALB/c mice showed bacterial burden in the lung, liver, and spleen, which was significantly diminished in the animals that were immunized with live attenuated *Salmonella* expressing the VapA protein; i.e., with χ3987-pYA3137*vapA* (data not shown). In agreement with our previous report [27], CFU recovery from the lung, liver, and spleen was 3- to 4-fold lower in the VapA-immunized mice than in the two control groups of mice, which received *Salmonella* χ3987-pYA3137 or PBS, respectively. These results confirm that immunization with attenuated *S. enterica* Typhimurium expressing VapA confers resistance to *R. equi* infection.

**Figure 1:**
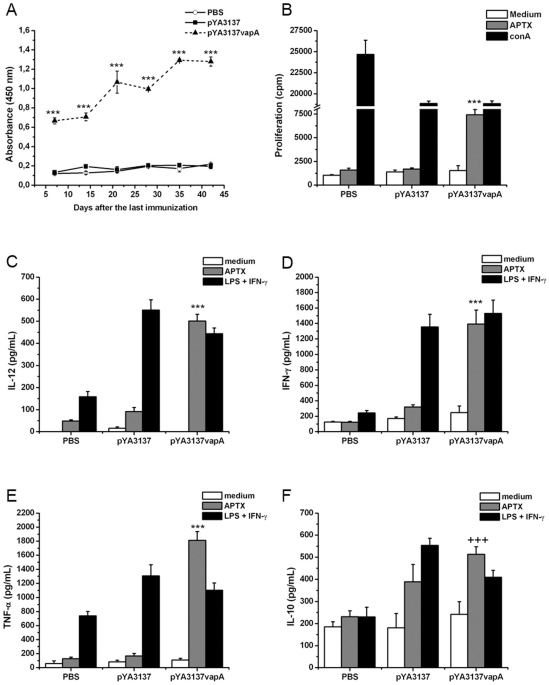
Humoral and cellular immune responses elicited by vaccination with *Salmonella* expressing the VapA protein. (A) Fecal IgA antibody response. Groups of BALB/c mice were orally immunized on days 0 and 14 with *S. enterica* Typhimurium χ3987-pYA3137*vapA* (closed triangles/dashed line), or inoculated with either *S. enterica* Typhimurium χ3987-pYA3137 (closed squares) or PBS (open circles). Fecal extracts were obtained on days 7, 14, 21, 28, 35, and 42 after the second immunization, for measurement of specific IgA by ELISA. The *R. equi* surface antigen (APTX) was used as the coating antigen (1 µg/mL). Results are expressed as the mean of OD values of five mice per group and are a representative experiment of three assays. ***p<0.001 compared to control groups. (B) Lymphocyte proliferative response. Spleen cells were harvested from immunized (pYA3137*vapA*) and control mice (pYA3137 or PBS) on day 14 after the last immunization and cultured in the presence of medium alone, APTX (5 µg/mL), or Concanavalin A (2 µg/mL) for 72 h. Cell proliferation was measured by [^3^H]-thymidine incorporation assay. Each bar represents the average of five mice per group ± SD and is representative of four independent experiments. ***p<0.001 compared to control groups. (C–F) Cytokines production. Whole spleen cells were collected from immunized (pYA3137*vapA*) and control mice (pYA3137 or PBS) on day 14 after the last immunization and cultured in the presence of medium alone, APTX (5 µg/mL), or LPS (1 µg/mL) plus IFN-γ (1 ng/mL) for 48 h. The concentration of IL-12 (C), IFN-γ (D), TNF-α (E), IL-10 (F), and IL-4 (not shown) cytokines in the supernatants of the cell cultures was measured by ELISA. Each bar represents the mean ± SD of triplicate samples and is a representative experiment of three assays. ***p<0.001 compared to the two control groups; ^+++^p<0.001 compared to PBS-inoculated mice.

In order to evaluate if, in addition to eliciting high levels of specific IgG circulating antibodies [27], oral immunization with live attenuated *Salmonella* expressing VapA would also induce humoral mucosal immunity, specific IgA was measured in the fecal extracts of mice. The IgA levels found in the immunized mice were at least 5-fold higher than those verified in the feces of control mice during the whole 42-day period that followed the second immunization (Fig. 1A), thus indicating that a strong and persistent mucosal response production was triggered by immunization with *S. enterica* Typhimurium expressing VapA. A higher production of IgA was also detected in the lung homogenates of immunized mice after infection with *R. equi*. Three- and 2-times higher production of IgA was detected in the lung of immunized mice compared with control mice, on days 2 and 8 post-challenge with *R. equi*, respectively (data not shown).

To investigate whether immunization with *S. enterica* Typhimurium expressing VapA could induce cell-mediated immunity against *R. equi*, spleen cells from immunized and control mice were assayed in terms of their antigen-specific proliferative response. A preparation of *R. equi* surface proteins (APTX) induced proliferation of spleen cells from immunized mice only (Fig. 1B), while a polyclonal stimulus (ConA) induced proliferation of cells from control and immunized mice. Intriguingly, proliferative response to ConA was significantly inhibited in animals that received χ3987-pYA3137, expressing VapA or not.

Together, these results indicate that VapA-immunization triggers mucosal-humoral response and systemic cell-mediated immunity.

### *R. equi* Antigen Stimulates the Production of Th1 Cytokines by Spleen Cells from Mice Vaccinated with *Salmonella* χ3987-pYA3137*vapA*

To characterize the cytokine production associated with inoculation of *Salmonella* χ3987-pYA3137*vapA*, cultures of spleen cells from mice of the immunized group and mice of the two control groups were stimulated with APTX. The positive control of stimulation was provided by LPS/IFN-γ, whereas medium alone was used as the negative counterpart. Spleen cells from mice of both control groups did not respond to APTX through cytokine production, whereas LPS/IFN-γ, as expected, markedly stimulated cytokines production by cells from mice that had been inoculated with *Salmonella* χ3987-pYA3137, expressing VapA or not (Fig. 1C-F). APTX only stimulated cells from mice immunized with *Salmonella* χ3987-pYA3137*vapA*, which produced IL-12, IFN-γ, and TNF-α in levels as high as those produced by cells stimulated with LPS/IFN-γ (Fig. 1C–E). Concerning IL-10, cells from mice immunized with *Salmonella* χ3987-pYA3137*vapA* furnished levels that were significantly higher than those produced by cells of the PBS control group. However, IL-10 levels produced by the former cells were similar to those provided by cells of mice of the second control group, which received *Salmonella* χ3987-pYA3137 (Fig. 1F). On the other hand, no IL-4 was detected in the spleen cells supernatant of the immunized mice (data not shown).

**Figure 2:**
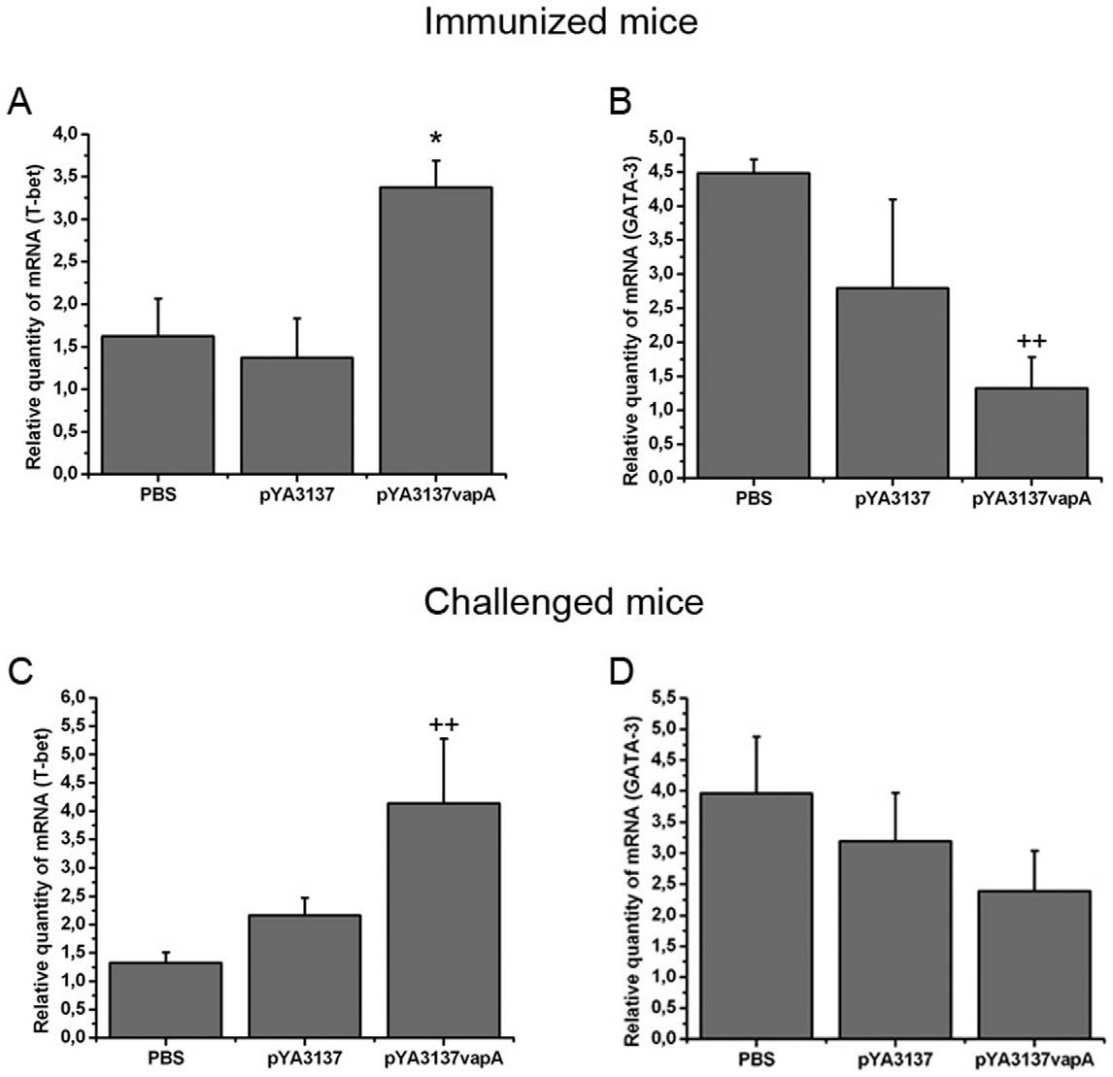
Transcription levels of T-bet and GATA-3 mRNA in spleen cells of mice vaccinated with *Salmonella* expressing the VapA protein. (A–B) Groups of BALB/c mice were orally immunized on days 0 and 14 with *S. enterica* Typhimurium χ3987-pYA3137*vapA*, or inoculated with either *S. enterica* Typhimurium χ3987-pYA3137 or PBS. On day 14 after the second immunization, spleen cells were harvested and the total RNA was extracted and assessed by real-time PCR. (C–D) Immunized (pYA3137*vapA*) and control (pYA3137 or PBS) mice were intravenously challenged with 4×10^6^ CFU of virulent *R. equi* strain 14 days after the last immunization. Spleen cells were collected 8 days post-infection, and the total RNA was extracted and assessed by real-time PCR. The cDNA contents were normalized on the basis of predetermined β-actin levels. Data are mean ± SD of triplicate samples in one of the three similar experiments. *p<0.05 compared to the two control groups; ^++^p<0.01 compared to the PBS control group.

Since T-bet and GATA-3 are well characterized transcription factors for differentiation of Th1 and Th2 cells, respectively, the mRNA levels for both transcription factors was analyzed. In agreement with the high production of cytokines related to Th1 skewed immunity, spleen cells of immunized mice showed higher mRNA levels for T-bet and lower for GATA-3, compared with the levels of the two control groups (Fig. 2A and 2B).

### The Th1 Immunity Induced by Vaccination with *Salmonella* χ3987-pYA3137*vapA* Is Sustained Following *R. equi* Challenge

**Figure 3:**
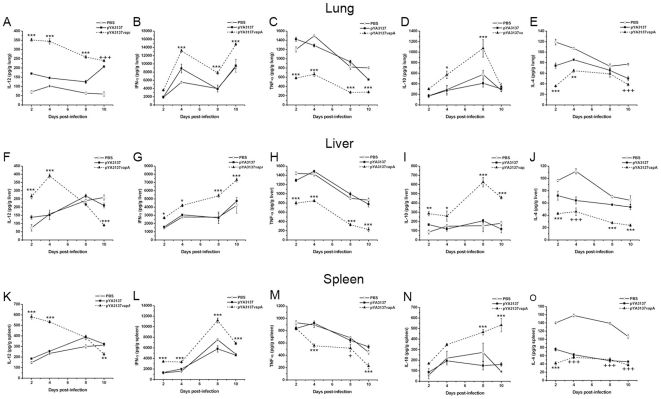
Cytokine levels in the organs of mice after *R. equi* infection. BALB/c mice were orally immunized on days 0 and 14 with *S. enterica* Typhimurium χ3987-pYA3137*vapA* (closed triangles/dashed line), or inoculated with either *S. enterica* Typhimurium χ3987-pYA3137 (closed squares) or PBS (open circles). All mice were intravenously challenged with 4×10^6^ CFU of *R. equi* ATCC33701 14 days after the last immunization. The lung (**A–E**), liver (**F–J**), and spleen (**K–O**) were harvested 2, 4, 8, and 10 days post-infection and homogenized for IL-12 (**A**, **F**, and **K**), IFN-γ (**B**, **G**, and **L**), TNF-α (**C**, **H**, and **M**), IL-10 (**D**, **I**, and **N**), and IL-4 (**E**, **J**, and **O**) detection. Results are expressed as means of five mice per group ± SD and are representative of three experiments. *p<0.05, **p<0.01, and ***p<0.001 compared to the two control groups; and **^+^**p<0.05, **^++^**p<0.01, and **^+++^**p<0.001 compared to the PBS control group.

To investigate the effect of *R. equi* infection on the pattern of immune response induced by VapA-immunization, the cytokines levels in organs of mice that had been intravenously challenged with virulent *R. equi* were examined two weeks after the last immunization. Groups of mice were sacrificed in different periods (2 to 10 days) post-challenge, and the time-course curves of individual cytokine content in the lungs, liver, and spleen are represented in Fig. 3. As a general rule, there was no significant difference in the cytokines levels detected in the organs of the two control groups of mice during the period of observation (10 days post-infection). As an exception, IL-4 levels in the organs of the χ3987-pYA3137 control group were nearer to the levels detected in the *Salmonella* χ3987-pYA3137*vapA* immunized mice, compared with the levels detected in the control group that received PBS, a fact that was remarkable in the spleen. A high IL-12 content was early detected in the organs of immunized mice and was sustained at least for 4 days in the spleen and liver, and 8 days in the lung. In the organs of non-immunized mice, a tendency to increasing IL-12 content was observed only later (8–10 days after challenge).

High IFN-γ levels were detected after challenge in all examined organs of immunized mice. Although in all groups of mice the IFN-γ content increased progressively after *R. equi* challenge, the levels attained by the immunized group were significantly higher than those of control groups in the whole observation period.

The TNF-α content was markedly lower in the lung and liver of immunized mice during the whole observation period. Less significant differences were detected in the spleen on days 2 and 8 post-challenge.

The IL-10 content was low and stable in the organs of control mice, while in immunized animals the IL-10 content had augmented progressively, reaching the highest levels by day 8 and 10 post-challenge in the lung and liver, and in the spleen, respectively.

IL-4 concentrations were persistently lower in the organs of immunized mice compared with the PBS control group, but sometimes similar to the IL-4 concentrations in the organs of the χ3987-pYA3137 control group, especially in the spleen (Fig. 3E, J, and O).

Eight days after challenge, we found that the mRNA transcription level of T-bet was higher in the spleen cells of immunized mice compared with the PBS control group and not different from the χ3987-pYA3137 control group (Fig. 2C). The expression of GATA-3 mRNA in the spleen tissue was not significantly different among the three groups of mice (Fig. 2D).

### Vaccination with *Salmonella* χ3987-pYA3137*vapA* Enhances NO and H_2_O_2_ Production after *R. equi* Challenge

Because the secretion of high IFN-γ levels, which is the signature cytokine of Th1 cells, activates phagocytes to kill microbes by stimulating the generation of reactive oxygen species and reactive nitrogen intermediates, the levels of nitric oxide and hydrogen peroxide were measured in the lung, liver, and spleen of immunized mice in several periods following *R. equi* infection.

**Figure 4:**
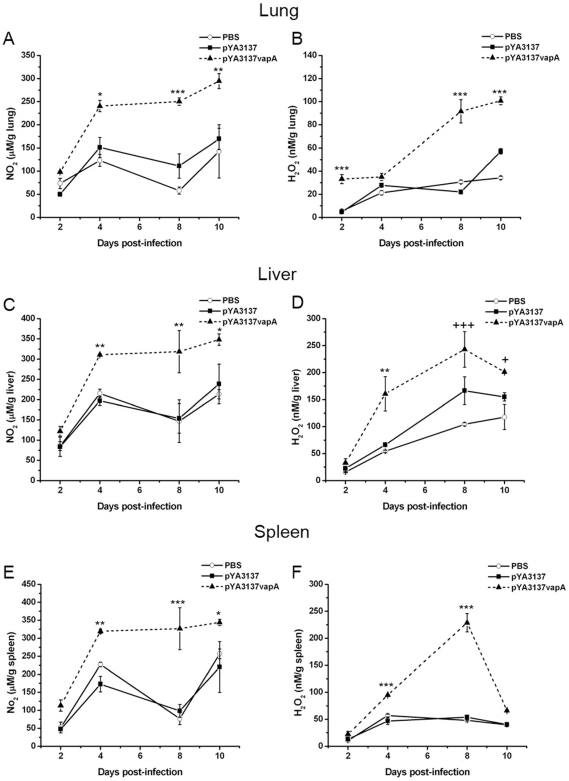
Nitrite and hydrogen peroxide production in the organs of mice following *R. equi* infection. BALB/c mice were orally immunized on days 0 and 14 with *S. enterica* Typhimurium χ3987-pYA3137*vapA* (closed triangles/dashed line), or inoculated with either *S. enterica* Typhimurium χ3987-pYA3137 (closed squares) or PBS (open circles). All mice were intravenously challenged with 4×10^6^ CFU of *R. equi* ATCC33701 14 days after the last immunization. On days 2, 4, 8, and 10 after infection, the lung, liver, and spleen were harvested and homogenized for NO (**A**, **C**, and **E**) and H_2_O_2_ (**B**, **D**, and **F**) detection. The endogenous H_2_O_2_ release was measured by the horseradish peroxidase-dependent phenol red oxidation methods, whereas the nitrite levels were measured by Griess reaction. Data are expressed as means of five mice per group ± SD and are representative of three separate experiments. *p<0.05, **p<0.01, and ***p<0.001 compared to the two control groups.

Significantly higher NO levels were detected in the organs of immunized mice compared with the control groups on days 4, 8, and 10 following *R. equi* infection (Fig. 4A, C, and E). There was no significant difference in the NO levels in the organs of the two control groups.

The H_2_O_2_ levels detected in the organs of immunized mice were also higher than those verified in the control groups, during the whole analysis period (Fig. 4B, D, and F). Maximum H_2_O_2_ production was verified 8 days following infection, when the levels in the lung and spleen of immunized mice were at least 3- and 4-fold higher, respectively, than those detected in the organs of control animals. Again, no significant differences were noted between the control groups.

### Splenic Lymphocyte Populations of *Salmonella* χ3987-pYA3137*vapA* Vaccinated Mice after *R. equi* challenge

**Figure 5:**
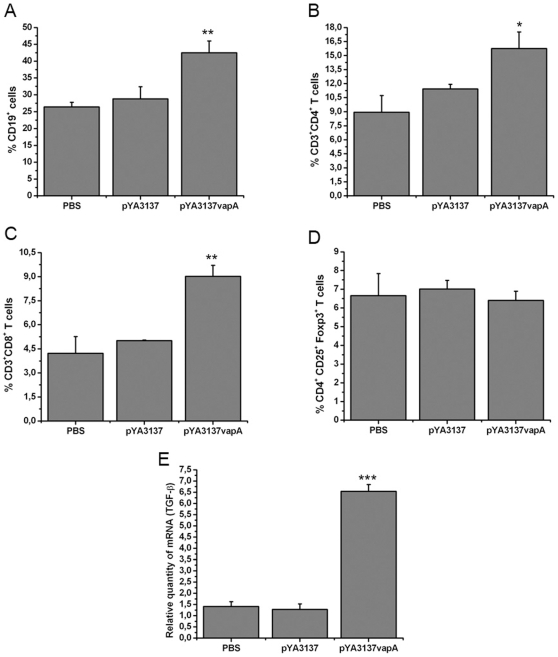
Alteration of lymphocyte subpopulation in the spleen of mice. BALB/c mice orally administered with *S. enterica* Typhimurium χ3987-pYA3137*vapA*, *S. enterica* Typhimurium χ3987-pYA3137, or PBS, on days 0 and 14, were intravenously challenged with 4×10^6^ CFU of virulent *R. equi* strain 14 days after the last immunization. Spleen cells were harvested 8 days post-infection and analyzed by flow cytometry. The cells were stained with specific anti-CD19 (**A**), anti-CD3 plus anti-CD4 (**B**), and anti-CD3 plus anti-CD8 (**C**) monoclonal antibodies. (**D**) CD4^+^ spleen T cells stained with specific anti-CD25 plus anti-Foxp-3 monoclonal antibodies. The average of the percentage of spleen cells positive for the respective CD markers in five samples is shown. Similar results were obtained in three independent experiments. (**E**) Total RNA was extracted from spleen cells harvested 8 days post-infection and assessed by real-time PCR for detection of TGF-β mRNA. The cDNA contents were normalized on the basis of predetermined β-actin levels. Data are mean ± SD of triplicate samples in one of the three similar experiments. *p<0.05, **p<0.01, and ***p<0.001 compared to the two control groups.

Because the cross-talk between T and B cells is of fundamental importance for the establishment of solid acquired immunity against facultative intracellular pathogens [30], we examined whether oral immunization with *Salmonella* χ3987-pYA3137*vapA,* followed by *R. equi* challenge, might influence the incidence of B, T helper, T cytotoxic and T regulatory lymphocytes among spleen cells. As shown in Fig. 5A, the rate of spleen cells expressing CD19, a pan-B cell marker, was higher in VapA-immunized than in control mice. The incidence of CD4^+^ and CD8^+^ T cells in spleen cells was also superior in immunized than in control mice (Fig. 5B and 5C). Collectively, these results suggest that both B-cells and CD4^+^- and CD8^+^-T cells can play roles in the protection against *R. equi* infection after oral vaccination with *Salmonella* χ3987-pYA3137*vapA*.

Because the organs of immunized mice with χ3987-pYA3137*vapA* showed a high IL-10 content and mild inflammatory reaction after *R. equi* infection [27], the incidences of T regulatory cells in the spleen cells of immunized mice and control groups were compared. Eight days after *R. equi* infection, CD4^+^ T spleen cells were purified, and the relative frequency of cells expressing CD25 and Foxp3 was determined. As shown in Fig. 5D, similar rates of CD4^+^CD25^+^Foxp3^+^ T cells were detected in the preparations of spleen cells of the immunized and control mice.

Transcription of TGF-β mRNA was also analyzed 8 days after *R. equi* infection, by real-time PCR. The spleen cells from immunized mice showed at least 4-fold larger TGF-β mRNA transcription levels than the control groups (Fig. 5E).

These results indicate that the higher IL-10 and TGF-β detection in *Salmonella* χ3987-pYA3137*vapA* immunized mice is not due to a higher frequency of CD4^+^CD25^+^Foxp3^+^ T cells.

### The *Salmonella* χ3987-pYA3137*vapA* Vaccinated Mice Are Long-Term Protected against *R. equi* Infection

To evaluate whether the immunization with *Salmonella* χ3987-pYA3137*vapA* induces a long-term protection against rodococcosis, vaccinated and control groups of mice were intravenously challenged with virulent *R. equi* strain 5 months after the last immunization. On day 8 post-challenge, the spleen and liver homogenates were evaluated in terms of bacterial counting and NO levels.

**Figure 6:**
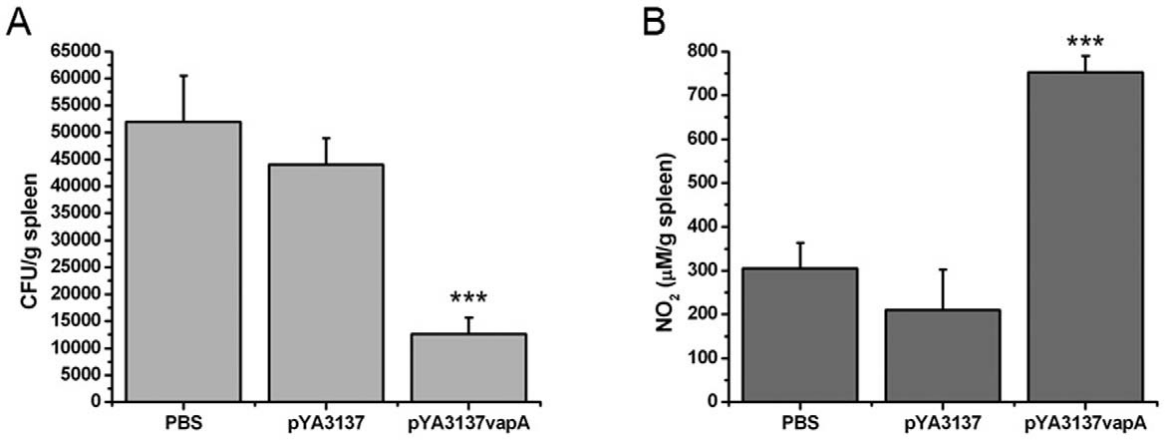
Recall immune response induced by immunization with *Salmonella* expressing the VapA protein. BALB/c mice were orally immunized with *S. enterica* Typhimurium χ3987-pYA3137*vapA*, or inoculated with either *S. enterica* Typhimurium χ3987-pYA3137 or PBS on days 0 and 14. Five months after the last immunization, all mice were intravenously challenged with 4×10^6^ CFU of virulent *R. equi*. On day 8 after infection, the spleen (**A** and **B**) and liver (not shown) were harvested and homogenized for bacterial burdens and NO quantification. Results are expressed as the mean of five mice per group ± SD. The experiment was performed twice with similar results. *p<0.05, **p<0.01, and ***p<0.001 compared to the control groups.

Significantly lower *R. equi* CFU were recovered from the tissues of immunized mice compared with the two control animal groups (Fig. 6A and data not shown). The two control groups had similar CFU. The number of CFU recovered from the spleen of immunized mice reached levels 4-fold inferior to those recovered from the spleen of control mice (Fig. 6A). Immunized mice produced a significantly higher amount of NO compared with the control groups (Fig. 6B). Taken together, these results indicate that immunization with *Salmonella* χ3987-pYA3137*vapA* induces a long-term protection against *R. equi* infection.

## Discussion

Vaccination with attenuated *S. enterica* Typhimurium χ3987 strain expressing the VapA antigen confers protection against *R. equi* infection. This had been demonstrated in oral immunized mice that, when challenged with *R. equi*, showed higher survival rates, higher bacterial clearance, and milder inflammatory response compared with the non-vaccinated mice [27]. In the present work we report that the immune response developed by the vaccinated mice with *S. enterica* Typhimurium expressing the VapA protein is featured by strong mucosal-humoral and systemic cell-mediated immunity. Besides that, we show that the vaccination confers a long-term protection against *R. equi* infection.

The occurrence of mucosal immunity in response to vaccination is indicated by the detection of high and persistent fecal levels of antigen-specific IgA. This fact may be a relevant consequence of the immunization procedure because secretory immunoglobulins constitute a frontline defense against several microorganisms [25], [26].

The specific cellular immune response developed after vaccination with *Salmonella* expressing VapA is revealed by the proliferation of the spleen cells of immunized mice in response to the *in vitro* stimulation with *R. equi* antigen. Because *R. equi* is a facultative intracellular pathogen, resistance to infection crucially depends on effective cell-mediated immunity [5], [14], [31], [32]. This requirement has been previously demonstrated by the severe pulmonary granulomas and failure to eliminate the bacteria presented by athymic nude mice infected with *R. equi* [33]. Other authors have already reported that vaccination with distinct antigens carried by attenuated *S. enterica* Typhimurium is associated with antigen-stimulated lymphoproliferative response [21], [34].

The present study shows that Th1 cytokines are intensely produced by vaccinated mice. Firstly, the levels of IL-12, IFN-γ, and TNF-α cytokines released *in vitro* by antigen-stimulated spleen cells from vaccinated mice are significantly higher than those produced by cells from mice of the non-vaccinated control groups. Secondly, tissues of the vaccinated mice challenged with *R. equi* contain higher IFN-γ levels than the tissues of mice belonging to the control groups, during the whole observation period. Interestingly, the tissue detection of IL-12 has been precocious, peaked at 2 and 4 days post-infection, but it declined thereafter, in a period when the vaccinated mice had already cleared the infection and no more lesions were observed in their lung, liver or spleen [27]. The induction of Th1 immunity by vaccination has been previously suggested to occur on the basis of the detection of high serum levels of IgG2a specific antibodies in oral immunized mice [27]. It is well established that during *R. equi* infection Th2-biased immunity is associated with disease development, while Th1-prone response affords infection control [7], [14], [17]–[19], [33], [35]. Beyond doubt, the Th1-cell immunity developed by the vaccinated mice fits with the protection conferred against rodococcosis. Previous reports have associated the utilization of attenuated *Salmonella* strains as vaccine vectors with the occurrence of strong and sustained Th1-immunity [34].

The activities exerted by Th1 cytokines are crucial for the elimination of intracellular pathogens, such as *R. equ*i [7], [14], [17]–[19], [33], [35], [36]. IL-12, released by activated dendritic cells and macrophages, induces differentiation of Th0 into Th1 cells. The release of IFN-γ by Th1 cells promotes macrophage effector activities, such as those mediated by nitric oxide and hydrogen peroxide, which have been detected in high levels in the lung, liver and spleen of vaccinated mice challenged with *R. equi.* As previously demonstrated, the synergistic action of nitric oxide and superoxide is crucial for the intracellular killing of *R. equi* [37]. Still regarding the Th1 cytokines, TNF-α is highly produced *in vitro* by the antigen-stimulated spleen cells of vaccinated mice, but is barely detected in the organs of vaccinated mice after *R. equi* challenge. This can be advantageous considering that TNF-α is a prototypical proinflammatory cytokine [38] whose overproduction is associated with the occurrence of host tissue injury [39].

IL-4, an essential cytokine for generation of Th2 responses [40], is not produced by the antigen-stimulated spleen cells of vaccinated mice. On the other hand, IL-4 is persistently detected in the organs of vaccinated mice after challenge with *R. equi*, albeit in concentrations significantly lower than those detected in the organs of non-vaccinated mice. With respect to IL-10, the antigen-stimulated spleen cells from vaccinated mice produce significantly higher levels of this cytokine than those afforded by the cells of mice belonging to the control group that received PBS instead of vaccine. However, the IL-10 levels in the spleen cells of the vaccinated mice are similar to those produced by the control mice that received *Salmonella* carrying the plasmid alone (without VapA expression). This strongly suggests that the vaccinal vector accounts for induction of IL-10 production. The IL-10 tissue levels in the organs of vaccinated mice augment progressively after *R. equi* challenge, reaching the maximum after day 8 post-challenge. IL-10 favors the balance between pathology and protection during diseases that course with inflammation. Such immunoregulatory role is due to the IL-10 ability of inhibiting the expression of the most inducible cytokines and secondary mediators that contribute to inflammation. Such a phenomenon may be responsible, in our study, for the decrease in TNF-α secretion, which is temporally coincident with the elevation of IL-10.

The induction of Th1-immunity by vaccination is evidenced by quantification of the expression of the transcription factors T-bet and GATA-3. Following vaccination, spleen cells of mice display a prominent mRNA expression of the Th1 transcription factor T-bet, in detriment of the Th2 transcription factor GATA-3, a finding that is consistent with the cytokines profile verified in the immunized animals. After challenge with *R. equi*, the vaccinated mice still present higher mRNA transcription level of T-bet than the non-vaccinated mice, while the levels of GATA-3 mRNA expression are not significantly different between cells from vaccinated and non-vaccinated mice.

Examination of the relative frequency of lymphocyte populations in the groups of mice challenged with *R. equi* reveals greater incidence of B lymphocytes (CD19^+^), CD4^+^ and CD8^+^ T cells in the vaccinated mice compared with the control. Thus, humoral and cellular mechanisms may be acting together, a hypothesis that is consistent with the previous demonstration that cooperation between humoral and cellular branches of immunity is necessary to protect the host against *R. equi* infection [13], [14], [16], [41], [42]. Concerning the T cell subpopulations, whilst CD4^+^ T cells generate the appropriate cytokine milieu to support resistance against *R. equi*, CD8^+^ T cells account for the destruction of the infected host cells [17], [43] as well as for the release of the bacteria, which become accessible to the humoral effector mechanisms [44]. Besides that, through cytokine-mediated interactions with B lymphocytes, the CD4^+^ T cells contribute to the generation of high-affinity IgG antibodies, which are able to perform bacterial opsonization and complement activation. So the lymphocyte populations provide multiple effector mechanisms capable of eliminating the *R. equi* infection.

Because secretion of IL-10 and TGF-β is one mechanism through which T regulatory cells exert suppression of effector immune response, whether this cell population could be expanded in the spleen of vaccinated mice has been investigated. There is no difference between vaccinated and non-vaccinated mice in terms of the relative frequency of CD4^+^CD25^+^Foxp3^+^ T cells, although the transcription levels of TGF-β mRNA are significantly higher in the spleen cells from vaccinated mice. Nevertheless, TGF-β can also be produced by naive CD4^+^ T cells upon TCR stimulation [45], and the high IL-10 production induced by vaccination may also be attributed to a non-Treg source. Recent studies have shown that Th1 cell themselves can produce IL-10, which is essential for the inhibition of exaggerated Th1 cell responses during infection [46], [47]. It has also been reported that CD4^+^ T cells producing high amounts of IFN-γ are the source of IL-10 that inhibits the development of immunopathology upon *T. gondii* infection [47]. These T cells express the Th1 cell lineage transcription factor T-bet, but not the Treg cell marker Foxp3. In any case, we postulate that the high IL-10 levels associated with vaccination exert an important role in maintaining the control of the inflammatory reaction that follows *R. equi* infection. This control is also favored by the low TNF-α present in the organs of the vaccinated mice, compared with the control mice. As already mentioned, TNF-α often mediates the host tissue injury that occurs in exacerbated inflammation, while IL-10 downmodulates Th1 immunity and minimizes inflammatory tissue damage [39], [48], [49]. Therefore, our results provide enlightenment regarding our previous observation that, after being challenged with *R. equi*, vaccinated mice develop a milder inflammatory reaction than that observed in the control animals.

Conceptually, an effective vaccine must be able to induce a long-term memory against the heterologous antigen. We have verified that vaccination with *Salmonella* χ3987-pYA3137*vapA* induces a protection against *R. equi* infection with a minimal duration of 5 months after the last immunization. Compared with the non-vaccinated animals, a significantly lower number of bacteria are recovered from the spleen of the vaccinated mice, and their spleen cells release higher concentration of nitric oxide. These observations indicate that vaccinated mice are still immune late after vaccination.

In conclusion, the *Salmonella enterica* serovar Typhimurium χ3987-pYA3137*vapA* is endowed of properties that feature a good vaccine to prevent rhodococcosis: (1) it confers protection against *R. equi* infection; (2) it induces strong and specific mucosal, humoral, and cell-mediated Th1-responses against the heterologous antigen; (3) it generates an appropriate regulatory cytokine response, which allows for tissue integrity; and (4) it induces a long-term protection. The immunization of foals is being performed by our group, to investigate the efficiency of the vaccination in the species most frequently affected by *R. equi*.
